# Synthesis, Hydrophilicity and Micellization of Coil-Brush Polystyrene-*b*-(polyglycidol-*g*-polyglycidol) Copolymer—Comparison with Linear Polystyrene-*b*-polyglycidol

**DOI:** 10.3390/polym14020253

**Published:** 2022-01-08

**Authors:** Mariusz Gadzinowski, Maciej Kasprów, Teresa Basinska, Stanislaw Slomkowski, Łukasz Otulakowski, Barbara Trzebicka, Tomasz Makowski

**Affiliations:** 1 Centre of Molecular and Macromolecular Studies, Polish Academy of Sciences, H. Sienkiewicza 112, 90-363 Lodz, Poland; mariuszg@cbmm.lodz.pl (M.G.); basinska@cbmm.lodz.pl (T.B.); tomekmak@cbmm.lodz.pl (T.M.); 2 Centre of Polymer and Carbon Materials, Polish Academy of Sciences, M. Curie-Skłodowskiej 34, 41-819 Zabrze, Poland; mkasprow@cmpw-pan.edu.pl (M.K.); lotulakowski@cmpw-pan.edu.pl (Ł.O.); btrzebicka@cmpw-pan.edu.pl (B.T.)

**Keywords:** Coil-Brush block copolymer, polystyrene-polyglycidol micellization, wettability of copolymer film

## Abstract

In this paper, an original method of synthesis of Coil-Brush amphiphilic polystyrene*-b*-(polyglycidol-*g*-polyglycidol) (PS-*b*-(PGL-*g*-PGL)) block copolymers was developed. The hypothesis that their hydrophilicity and micellization can be controlled by polyglycidol blocks architecture was verified. The research enabled comparison of behavior in water of PS-*b*-PGL copolymers and block–brush copolymers PS-*b*-(PGL-*g*-PGL) with similar composition. The Coil-Brush copolymers were composed of PS-*b*-PGL linear core with average *DP*_n_ of polystyrene 29 and 13 of polyglycidol blocks. The *DP*_n_ of polyglycidol side blocks of coil–*b*–brush copolymers were 2, 7, and 11, respectively. The copolymers were characterized by ^1^H and ^13^C NMR, GPC, and FTIR methods. The hydrophilicity of films from the linear and Coil-Brush copolymers was determined by water contact angle measurements in static conditions. The behavior of Coil-Brush copolymers in water and their critical micellization concentration (CMC) were determined by UV-VIS using 1,6-diphenylhexa-1,3,5-trien (DPH) as marker and by DLS. The CMC values for brush copolymers were much higher than for linear species with similar PGL content. The results of the copolymer film wettability and the copolymer self-assembly studies were related to fraction of hydrophilic polyglycidol. The CMC for both types of polymers increased exponentially with increasing content of polyglycidol.

## 1. Introduction

Amphiphilic block copolymers (BCPs) have found numerous applications in various fields of science and branches of industry such as drug delivery carriers [1,2,3,4,5], bioactive agents carriers [6,7], tissue engineering materials [8], electrochemical sensors [9], polymer blends [10], polymer membranes [11], nanoreactors with embedded enzymes [12], enhanced-performance supercapacitors [13], anti-bacterial and anti-protein fouling coatings of various materials [14,15,16], surfactants systems for stabilization of various emulsions [17], and polymer solar cells [18]. The final application of copolymers is closely related to the chemical structure, architecture of macromolecules and properties of blocks components. Amphiphilic block copolymers undergo self-assembly, which very often leads to the formation of hierarchical periodic nanostructures. To date, BCPs composed of co-blocks with various ratio of molar mass of individual segments are used for the preparation of various (hierarchical) structures such as spherical, cylindrical, vesicular, lamellar, bicontinuous gyroids, etc. [19,20,21].

In spite of the same chemical composition, amphiphilic copolymers may significantly differ in architecture. In turn, the architecture affects copolymers organization in nano- or microstructures and impacts on their final application.

Recently, amphiphilic block copolymers with Coil-Brush (also named coil–bottle brush or coil–comb) architecture were synthesized. For instance, polystyrene coil and brush composed of methyl and lauryl acrylates and oligo(ethylene glycol) methyl ether acrylate were used to produce hierarchically structured nanocomposites [22]. Other studies revealed that self-assembly of amphiphilic copolymers composed of poly(N-methylglycine)-*b*-poly(N-decyl glycine) leads to formation of nano- or microsized particles with various shape and morphology from long worm-like structures to 2D sheets, depending on N-decyl glycine content in copolymer [23].

The computer simulation of coil–comb diblock copolymers was used for comparison of disordered to ordered phase transitions. It was calculated that for some coil–coil and Coil-Brush compositions, these phase transitions were significantly different. Generally, it was found that the comb copolymer with longer main chain and shorter side chains is more resistant to the disordered–ordered phase transition [24].

Other studies were undertaken to synthesize a series of Coil-Brush block copolymers (CBCPs) such as: polystyrene-*b*-(poly(2-(2-bromopropionyloxy)styrene)-*g*-poly(methyl methacrylate)) (PS-*b*-(PBPS-*g*-PMMA)) and polystyrene-*b*-(poly(2-(2-bromopropionyloxy)ethyl methacrylate)) (PS-*b*-(PBPEA-*g*-PMMA)). It was found that the mentioned Coil-Brush copolymers formed nanostructures via microphase separation [25].

As mentioned before, the architecture of amphiphilic copolymers affects the size, shape, and morphology of formed particles (often micelles). It was reported that amphiphilic Coil-Brush di- and tri-block copolymers with hydrophobic polystyrene block and hydrophilic block(s) composed of poly(acrylic acid) grafted on modified polystyrene self-assemble in dioxane/water mixed solvent to form nanoparticles [26]. The morphology and size of the obtained objects depended on the 1,4-dioxane/water ratio and the architecture of the copolymers. For instance, it was noticed, that shape and size of the micelles were determined by the location of the side poly(acrylic acid) chains in the polystyrene backbone. In addition, in the case of polystyrene-based anionic diblock copolymers the lower fraction of water in mixed solvent favoured formation of micelles, whereas higher fraction of water resulted in production of micellar aggregates with hydrodynamic diameters in the range from 500 to 1000 nm [26].

Similar formation of micelles and micelles aggregates was observed for linear amphiphilic copolymer PS-*b*-PGL in water/dioxane mixtures. It was shown that morphology of obtained aggregates was dependent on water content and hydrophobic to hydrophilic balance of the blocks in copolymer [27].

Amphiphilic block copolymers very often contain poly(ethylene oxide) blocks used as the hydrophilic segments. These blocks are usually synthesized by anionic ring-opening polymerization of ethylene oxide [28,29]. However, for the synthesis of block copolymers with branched or brush architecture of one or more blocks, the monomer containing additional functional side group(s) is required. The good candidate for such a monomer is glycidol, which, after blocking hydroxyl groups, could be polymerized by the anionic ring-opening polymerization. Deprotection of hydroxyl groups leads to the linear polyglycidol with hydroxymethylene side group in each repeating polymeric unit [30,31]. This approach (blocking hydroxyl groups–polymerization–deblocking) was used in our earlier studies on synthesis and micellization of polystyrene-*b*-polyglycidol (PS-*b*-PGL) with (hydrophobic coil)-*b*-(hydrophilic coil) architecture [32].

In the literature, there are many types of copolymers with hydrophilic and hydrophobic blocks described. One very good example is a review by L.I. Atanase et al. discussing micellization properties of synthetic and polysaccharides-based graft copolymers in aqueous media [33]. However, according to our best knowledge, there are no reports on studies comparing properties of simple amphiphilic diblock copolymers with the same, or very similar, chemical composition in the literature. Such studies could verify for the first time the hypothesis that the copolymer chain architecture alone affects copolymer properties and show how large this effect may be.

For our studies, we selected amphiphilic copolymers with polystyrene-*b*-(polyglycidol-*g*-polyglycidol) (PS-*b*-(PGL-*g*-PGL)) (hydrophobic coil)-*b*-(hydrophilic brush) and compare their micellization and copolymer film hydrophilicity with corresponding properties of PS-*b*-PGL (hydrophobic coil)-*b*-(hydrophilic coil) copolymers. For both copolymer classes, the *DP*_n_ of the polystyrene block was the same at 29 (the same polystyrene sample with the anionic end-groups was used as macroinitiator for synthesis of polyglycidol containing blocks). For both classes, the number of polyglycidol units in macromolecules varied in the range from 13 to 136.

**Scheme 1:**
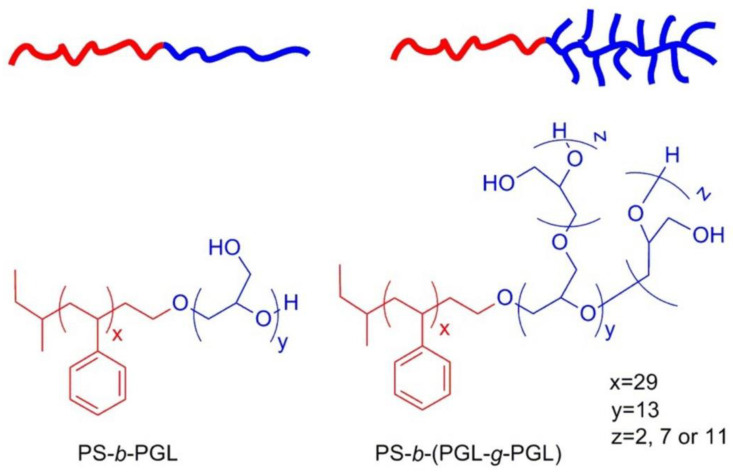
Planned structures of PS-*b*-PGL and PS-*b*-(PGL-*g*-PGL) block copolymers.

The structures of initial linear PS-*b*-PGL and Coil-Brush PS-*b*-(PGL-*g*-PGL) diblock copolymers are shown in Scheme 1.

## 2. Materials and Methods

### 2.1. Materials

Styrene (Sigma-Aldrich, Poznan, Poland) was kept in a deaerated ampoule over calcium hydride removing the monomer stabilizer. The purified styrene was distilled using a vacuum line and stored at 4 °C. Toluene (Chempur, Piekary Slaskie, Poland) was purified by stirring it with added sulfuric acid, neutralization with water solution of NaHCO_3_, and drying over MgSO_4_. Final drying was carried out by boiling over sodium under reflux. The needed amounts of toluene were distilled immediately before being used. Ethylene oxide (Fluka) was dried over calcium hydride in a deaerated ampoule equipped with a Teflon^®^ stopcock. The dry monomer was distilled using a vacuum line and stored at 4 °C before being used. Tetrahydrofuran (THF, Chempur, Piekary Slaskie, Poland) was purified from traces of peroxides by addition of potassium hydroxide and keeping this mixture for a few days. Then, THF was distilled using a vacuum line and stored over metallic sodium. Required amounts of THF were distilled to ampoules containing a sodium–potassium alloy for further storage. 1-Ethoxyethyl glycidyl ether (GLB) was synthesized as described before [34,35]. Then, GLB was distilled using a vacuum line and dried over calcium hydride. *Sec*-butyllithium (1.4 M solution in cyclohexane, Sigma-Aldrich), 1,6-diphenylhexa-1,3,5-trien (DPH) (Sigma-Aldrich), aluminum chloride hexahydrate, 1,4-dioxane, and metallic potassium (Sigma-Aldrich) were used as received. 1,4-dioxane, dimethylformamide (DMF) (Sigma-Aldrich), were used as received. The water used to obtain the polymer solutions was purified using a commercial ion exchange system (Hydrolab, Straszyn, Poland).

### 2.2. Methods

#### 2.2.1. Synthesis of PS-*b*-(PGL-*g*-PGL) Copolymers

The polyglycidol grafts on PS-*b*-PGL copolymer were prepared by polymerization of glycidol with protected hydroxyl group (GLB) initiated with hydroxyl groups of PS-*b*-PGL converted to alkoxide anions. The exemplary synthesis is described below (synthesis of PS-*b*-(PGL-*g*-PGL)1 sample; characteristics of copolymers are given in Table 1). The PS-*b*-PGL1 diblock copolymer (1.00 g, 0.000244 mol) was first dissolved in 5 mL of dry 1,4-dioxane and activated by 24 h at r.t. with freshly prepared potassium mirror in the deaerated glass reactor consisting of two connected vials. The resulting potassium polyalkoxide solution was poured into deaerated co-vial containing 4 mL of dry DMSO and 1.401 g (0.00960 mol) of GLB. Polymerizations were carried out at 45 °C for 4 days. Thereafter, it was terminated by adding excess of formic acid solution. The resulting copolymer was precipitated into warm deionized water. The crude viscous product was dried in vacuum, then dissolved in 1,4-dioxane and precipitated into methanol. Deprotection of hydroxyl groups yielding the PS-*b*-(PGL-*g*-PGL)1 copolymer was performed in the following way: 2.0 g of copolymer was dissolved in 2 mL of 1,4-dioxane. To this solution, 10 mL of methanol was added dropwise until the mixture become slightly opaque. Then, 10 mg of AlCl_3_·6H_2_O was added and the mixture was stirred at 40 °C for 3 h. The copolymer was dialyzed against 10^−2^ mol/L HCl and subsequently dialyzed against water using SERVA SpectraPor dialyzing tube with MWCO (molar mass cut-off) 1000 g/mol. The final product was lyophilized.

#### 2.2.2. Micelle Formation

The self-assembling process of Coil-Brush and block copolymers was performed using dialysis method, which is one of the standard methods of preparing micelles [35]. In our earlier studies, we observed that aggregates of PS-*b*-PGL copolymers produced by dialysis method do not disintegrate, even when samples were diluted with water far below CMC and stored for several days. Therefore, any micelles and aggregates observed by DLS were formed during the dialysis step, and their proportion did not change thereafter. In order to obtain micelles, a series of polymer solutions with concentrations from 2.018 to 0.00488 g/L for PS-*b*-(PGL-*g*-PGL)1, 4.97 to 0.026 g/L for PS-*b*-(PGL-*g*-PGL)2, and 10.064 to 0.098 g/L for PS-*b*-(PGL-*g*-PGL)3 in DMF were made, and then samples of 3 mL each were dialyzed (MWCO 1000 g/mol) against water for 5 days, replacing the solvent daily. In the following step, the dialyzed solutions were transferred into weighted vials in order to determine final concentration of copolymer.

### 2.3. Characterization

#### 2.3.1. ^1^H and ^13^C NMR Spectra

^1^H and ^13^C NMR spectra of intermediate products obtained after each step of synthesis and of the final Coil-Brush PS-*b*-(PGL-*g*-PGL) block copolymer were registered using a Bruker (200 MHz) NMR spectrometer. The PS-OH sample and block copolymers with protected hydroxyl groups were dissolved in CDCl_3_, whereas diblock copolymers with free hydroxyls were dissolved in DMSO.

#### 2.3.2. Determination of M_n_, M_w_/M_n_, and dn/dc of PS-*b*-(PGL-*g*-PGL) Block Copolymers by GPC-MALLS

Gel permeation chromatography (GPC) measurements of the final PS-*b*-(PGL-*g*-PGL) copolymers were performed at 45 °C in N,N-dimethylformamide (DMF) with the addition of 5 mmol/L LiBr at a nominal flow rate of 1 mL/min. The chromatography system consisting of an isocratic pump Agilent 1260, multiangle light scattering detector (DAWN HELEOS II, Wyatt Technology, Santa Barbara, CA, USA λ = 658 nm), spectrophotometric UV/Vis detector Agilent 1260, refractive index detector (SEC-3010, WGE Dr. Bures), Dallgow-Doeberitz, Germany and a column system (PSS gel GRAM guard and three columns PSS GRAM 100 Å, 1000 Å and 3000 Å). The molar mass and dispersity were evaluated using ASTRA 7.3.1.9 software from Wyatt Technologies. The refractive index increments (dn/dc) were measured separately for brush copolymers. The value of refractive index increments were measured using refractometric detector SEC-3010 dn/dc at wavelength λ = 620 nm. The data were processed using the BI-DNDCW software. For calibration of detector, the series of polystyrene (10 000 g/mol) solutions in DMF with concentration ranging from 0.4 to 4.4 g/L were prepared. The refractive index increments values were 0.1156 mL/g for PS-*b*-(PGL-*g*-PGL)1, 0.0939 mL/g for PS-*b*-(PGL-*g*-PGL)2, and 0.0882 mL/g for PS-*b*-(PGL *g*-PGL)3. The five solutions of each copolymer dissolved in DMF, with concentration in the range of 0.5 g/L to 8.5 g/L were prepared in 5 mL volumetric flasks. The measurements were carried out at 45 °C and each copolymer solution was measured minimum 3 times.

#### 2.3.3. FTIR of Coil-Brush Copolymers

Fourier transform infrared spectra (FTIR) of the PS-*b*-(PGL-*g*-PGL) copolymers (solvent-free) were measured by attenuated total reflectance (ATR) technique using a Nicolet 6700 spectrometer equipped with a DGTS detector. Each spectrum was a sum of 64 scans at resolution of 2 cm^−1^.

#### 2.3.4. Determination of Copolymers CMC Using Spectrophotometric Method

For spectrophotometric determination of CMC, to each copolymer solution (1.5 mL volume) 15 μL of 0.6 mM of DPH in methanol was added and stored overnight in the dark. Finally, the absorbance spectra were registered using Specord200 Plus (Analytik, Jena, Germany) with Peltier cell temperature control. Measurements were carried out in 1.5 mL quartz glass measuring cuvette at 20 °C, stabilizing each sample for 120 s. Every sample was measured three times and absorbance values were averaged. The difference of absorbance value corresponding to each copolymer concentration was determined by subtraction of absorbance at λ = 400 nm from absorbance at λ = 382 nm (A382–A400). The CMC values were evaluated from plots of A382–A400 as a function of copolymer concentration. For each plot, the CMC value was taken as the point of intersection of the tangents of the curve.

#### 2.3.5. Determination of CMC, Hydrodynamic Diameters of PS-*b*-(PGL-*g*-PGL) Particles, and Diameter Dispersity Values by the Dynamic Light Scattering (DLS) Method

The hydrodynamic diameter (*D*_h_) and diameter dispersity were measured for particles suspensions prepared in a similar way as in the case of CMC measurements by spectrophotometric method (however, in absence of DPH). The measurements were performed using a Brookhaven BI-200 Goniometer with vertically polarized incident light of wavelength λ = 632.8 nm (He-Ne laser, 35 mW) equipped with a Brookhaven BI-9000 AT digital autocorrelator. The autocorrelation functions were analyzed using the constrained regularized CONTIN method providing distributions of relaxation rates (Γ). The apparent *D*_h_ was determined from the Stokes–Einstein Equation (1):

*D*_h_ = kT/3πηD (1)

where k denotes Boltzmann constant and η water viscosity at temperature T and D diffusion coefficient. Dispersity of particles diameters was calculated using Equation (2)

Dispersity = μ_2_/Γ^2^ (2)

where Γ is the average relaxation rate and μ_2_ is the second moment. CMC values were determined similarly as in the case of measurements by spectrophotometric method, namely, from the plots of scattering intensity (expressed in kilo counts per second) as function of copolymer concentration. CMC was taken as copolymer concentration corresponding to crossing point of tangent lines to experimental points.

#### 2.3.6. Water Contact Angle Measurements of PS-*b*-PGL and PS-*b*-(PGL-*g*-PGL) Films Deposited on Glass Plates

Prior to copolymers deposition, the glass slides were degreased by incubation in a mixture of isopropanol/KOH for 2 h and repeated washing with deionized water. Then, 0.2 g samples of each linear and Coil-Brush copolymer dissolved in 0.5 mL DMF were cast on the glass slides. Thereafter, glass slides with deposited copolymer solutions were dried at 75 °C for 3 h. Thickness of the films was 13 ± 2 μm. The films’ surface roughness (root mean square (RMS) of a surface height variation) was determined by atomic force microscopy (AFM). RMS measurements were carried on using the Flex Axiom Nanosurf apparatus with a C3000 controller (NanosurfAG, Liestal, Switzerland). The experiments were carried out in Dynamic Force Mode (Tapping Mode). The analysis was performed using probes (HI’RES-C14/CR-AU µmasch) with a typical spike radius 1 nm, a spring constant of 5 N/m, and a resonance frequency of 160 kHz. The images were recorded with a resolution of 512 × 512 data points. Image analysis and RMS measurements were carried out using a Scanning Probe Image Processor (SPIP) by Image Metrology, Hørsholm, Denmark. Roughness (RMS parameters) of films prepared from copolymers were PS-*b*-PGL2 (RMS = 19.4 nm; PS-*b*-PGL4 (RMS = 18.7 nm); PS-*b*-(PGL-*g*-PGL)1 (RMS = 18.3 nm); and PS-*b*-(PGL-*g*-PGL)3 (RMS = 16.5 nm). The films were conditioned at r.t. in a closed chamber containing drying agent. Finally, the contact angles on each film were measured and denoted as “on dry film”. In the following step, the same films were incubated for 48 h in an environmental chamber at r.t. in air saturated with water vapors. Water contact angles were measured on films immediately after their withdrawal from the environmental chamber and denoted as “on a wet film”). The measurements of water contact angles were performed using a Phoenix-300 goniometer (SEO Surface Electro Optics, Kwonsungu, Suwon, Korea). The measurements of water contact angles were carried out using 5 μL drops of deionized water deposited on the copolymer films. The average values of ten independent measurements were registered.

#### 2.3.7. Cryo-TEM of Particles Obtained from PS-*b*-(PGL-*g*-PGL) Copolymers

Cryogenic Transmission Electron Microscopy (cryo-TEM) images were obtained using a Tecnai F20 X TWIN microscope (FEI Company, Hillsboro, OR, USA) equipped with field emission gun, operating at an acceleration voltage of 200 kV. Images were recorded on the Gatan Rio 16 CMOS 4k camera (Gatan Inc., Pleasanton, CA, USA) and processed with Gatan Microscopy Suite (GMS) software (Gatan Inc., Pleasanton, CA, USA). Specimen preparation was completed by vitrification of the aqueous solutions on grids with holey carbon film (Quantifoil R 2/2; Quantifoil Micro Tools GmbH, Großlöbichau, Germany). Prior to use, the grids were activated for 15 s in oxygen plasma using a Femto plasma cleaner (Diener Electronic, Ebhausen, Germany). Cryo-samples were prepared by applying a droplet (3 μL) of the suspension to the grid, blotting with filter paper, and immediately freezing in liquid ethane using a fully automated blotting device Vitrobot Mark IV (Thermo Fisher Scientific, Waltham, MA, USA). After preparation, the vitrified specimens were kept under liquid nitrogen until they were inserted into a cryo-TEM-holder Gatan 626 (Gatan Inc., Pleasanton, CA, USA) and analyzed in the TEM at −178 °C.

## 3. Results and Discussion

### 3.1. Synthesis and Characterization of Polystyrene-b-(polyglycidol-g-polyglycidol)

The synthesis of a set of PS-*b*-(PGL-*g*-PGL) Coil-Brush copolymers consisted of the following steps: (i) anionic polymerization of styrene yielding polystyrene macroinitiator with hydroxyl end-groups (PS-OH); (ii) anionic polymerization of glycidol with hydroxyl groups blocked with 1-ethoxyethyl moieties (GLB) yielding PS-*b*-PGLB copolymer; (iii) deprotection of hydroxyl groups in PGLB; (iv) synthesis of oligo-GLB grafts from alkoxide active centers formed on PGL block; and (v) deprotection of hydroxyl groups yielding PS-*b*-(PGL-*g*-PGL). Polyglycidol grafts were polymerized on the linear copolymer PS-*b*-PGL1 with 29 of styrene repeating units and 13 glycidol units.

**Scheme 2:**
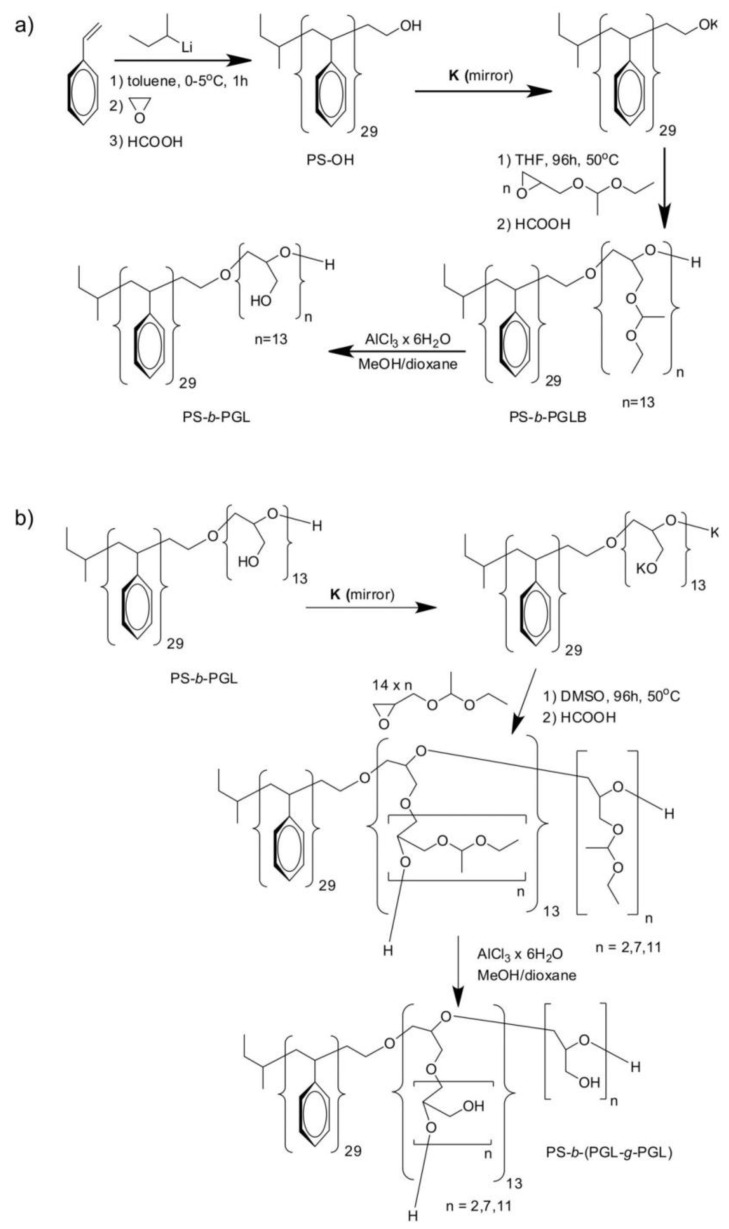
Reactions leading to planned copolymers: (**a**) linear PS-*b*-PGL and (**b**) Coil-Brush PS-*b*-(PGL-*g*-PGL) diblock copolymers.

The set of reactions involved in preparation of PS-*b*-(PGL-*g*-PGL) copolymer is presented in Scheme 2a,b.

Scheme 2a shows synthesis of linear PS-*b*-PGL block copolymer, whereas Scheme 2b presents further modification of the linear diblock copolymer yielding eventually copolymer PS-*b*-(PGL-*g*-PGL). The detailed description of synthesis of the diblock PS-*b*-PGL copolymers was described in our previous paper [27]. The description of synthesis of PS-*b*-(PGL-*g*-PGL) is described in the Materials and Methods section. The conversion of –OH groups into alkoxide initiating species and anionic polymerizations of PGLB were performed in the moisture-free deaerated apparatuses equipped with Teflon® stopcocks.

**Figure 1:**
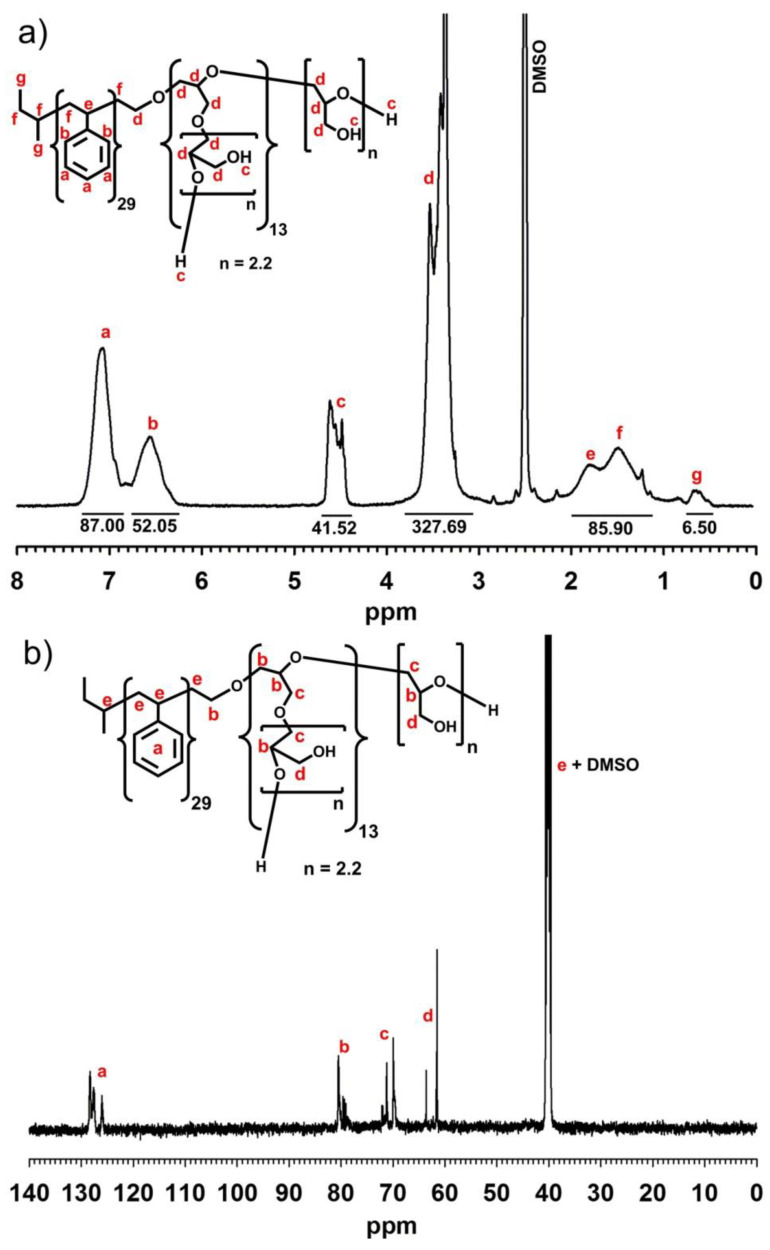
(**a**) ^1^H NMR and (**b**) ^13^C NMR spectra of PS-*b*-(PGL-*g*-PGL)1.

Figure 1a,b shows representative ^1^H and ^13^C NMR spectra of final PS-*b*-(PGL-*g*-PGL)1 copolymer sample (see Table 1) confirming its chemical structure.

In the ^1^H NMR spectrum, signals of protons in acetal side groups are absent, indicating that deprotection of hydroxyl groups blocked with ethylethoxy moieties in PGL side chains (see Scheme 1) was complete. For comparison, the ^1^H NMR spectrum of PS-*b*-(PGL-*g*-PGLB)1 copolymer with blocked hydroxyl groups is presented in Figure S1. Integration of the signals of hydroxyl end-groups’ of PGL observed in the ^1^H NMR spectra in the range 4.4–4.7 ppm (see the representative ^1^H NMR spectrum registered in DMSO, which is shown in Figure 1a) allowed determination of *DP*_n_ of the side PGL block. Similarly, chemical shifts in ^13^C NMR spectra of the signals of carbon atoms in PS-*b*-(PGL-*g*-PGL) copolymers confirmed their Coil-Brush structure. It should be noted that in ^13^C NMR spectra Figure 1b) the main signals b, c, and d are accompanied with the weaker ones corresponding to atoms of the end groups of the grafts. It is also worth stressing that polymerization degrees of all synthesized copolymers determined by ^1^H NMR were close to *DP*_n_ values calculated from compositions of polymerization mixtures (see Table 1).

The bands in the FTIR spectra of the Coil-Brush copolymers overlap and differ only in the intensity of the signals associated with the groups present in glycidol, which confirms the increase in the branch length (Figure S2). In particular, for copolymers with different numbers of units with hydroxyl groups, the absorption in the range 3600–3100 cm^−1^ (–OH stretching band) increased with increasing molar fraction of polyglycidol (f(PGL)) (see data in Table 1). Moreover, the strong band corresponding to –C-O stretching in alcohols (in the 1300–1000 cm^−1^ range) was noticed. Its intensity also increased with increasing fraction of PGL in copolymers.

**Figure 2:**
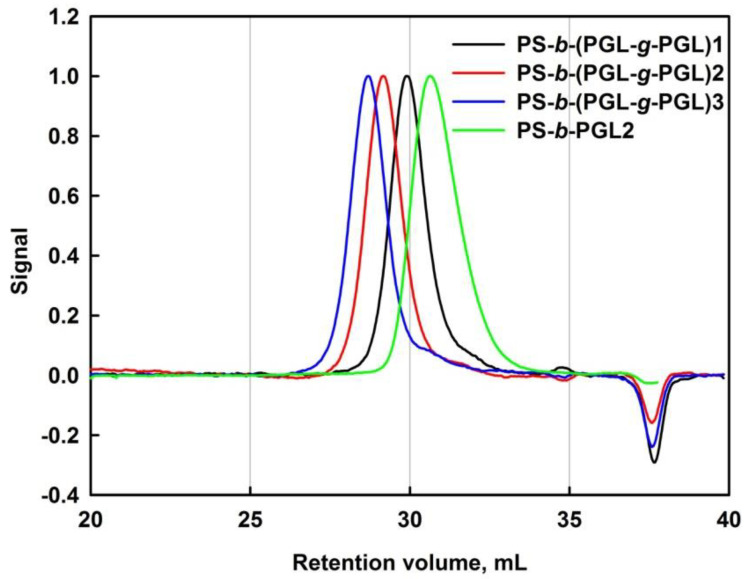
GPC-MALLS traces of linear PS-*b*-PGL1 core and set of PS-*b*-(PGL-*g*-PGL) Coil-Brush copolymers (DMF solvent).

Figure 2 presents GPC-MALS traces of the set of PS-*b*-(PGL-*g*-PGL) and PS-*b*-PGL copolymers.

It was found that the measured molar masses of all copolymers were very close to the ones calculated from composition of the polymerization mixture (see data in Table 1), regardless of the copolymer structure. These masses increased with the increasing number of PGL units in the side chains. It is worth mentioning that molar masses and dispersities were calculated using light scattering detection and dn/dc values determined for the copolymers’ chemical compositions (from ^1^H NMR) spectra and refractive index increments measured for individual blocks of prepared copolymers (Table 1) [36,37].

The GPC-MALLS signals for all analyzed polymers/copolymers (Figure 2 and Figure S3) were symmetrical and monomodal, indicating that all steps of diblock copolymers anionic polymerization proceeded in well controlled manner and the copolymers with low molar mass dispersity were synthesized.

Furthermore, it is worth noting that *DP*_n_ values of PGL side blocks in the set of PS-*b*-(PGL-*g*-PGL) copolymers determined from ^1^H NMR spectra and by GPC-MALLS were consistent. Thus, convergence of the results indicates that the anionic polymerization of glycidol grafting on the anionic centers of PGL core chains was controlled and complete. Moreover, it also indicates that deprotection of the side hydroxyl groups of all PGLB repeating units was successful and complete, as in the case of linear PS-*b*-PGLB [32]. Thus, elaborated method of manufacturing amphiphilic PS-*b*-PGL copolymers could be extended to synthesis of the copolymers with (PS coil)-*b*-(PGL brush) with precisely designed architecture and controlled length of each block. This is especially useful for studies of relations between copolymers’ architecture and their micellization behavior and association into more complex structures.

In principle, the method developed for synthesis of the PS-*b*-(PGL-*g*-PGL) copolymers could be extended to synthesis of similar copolymers containing polyglycidol with higher generation of branches with well controlled length.

### 3.2. Properties of PS-b-PGL and PS-b-(PGL-g-PGL) Copolymer Films

Water contact angles on hydrophobic surfaces, such as of polystyrene, are usually above 90°. On the other hand, polyglycidol is hydrophilic, as its hydroxyl and ether groups form hydrogen bonds with water molecules. It is known that polyglycidol thick layer grafted surfaces are superhydrophilic and very good water absorbents. Their wetting angles are typically below 10° [38]. It was expected that hydrophilicity of the PS-*b*-PGL and PS-*b*-(PGL-*g*-PGL) copolymers would be determined by the molar contribution of the hydrophilic polyglycidol blocks (see Table 2) and by their spatial arrangement. In order to test hydrophilicity of copolymers, the water contact angle measurements in static conditions on copolymer thin films were performed. Surface roughness (RSM parameter) of these films (determined by AFM) varied from 16.5 to 19.4 nm (see Figure S4). It is worth noting, that all analyzed linear PS-*b*-PGL copolymers, as well as PS-*b*-(PGL-*g*-PGL)2 and PS-*b*-(PGL-*g*-PGL)3 Coil-Brush copolymers, formed smooth films after film casting and solvent (DMF) evaporation at 75 °C. Unfortunately, the film of Coil-Brush copolymer with the lowest molar fraction of PGL (f(PGL) = 0.515) had numerous cracks and folds which did not allow for reliable determination of wetting angle. Presumably, the very short PGL grafts (*DP*_n_ = 1.78 see data in Table 1) on polymer chains obstructed proper packing of the polymer blocks and formation of films with smooth surface.

**Figure 3:**
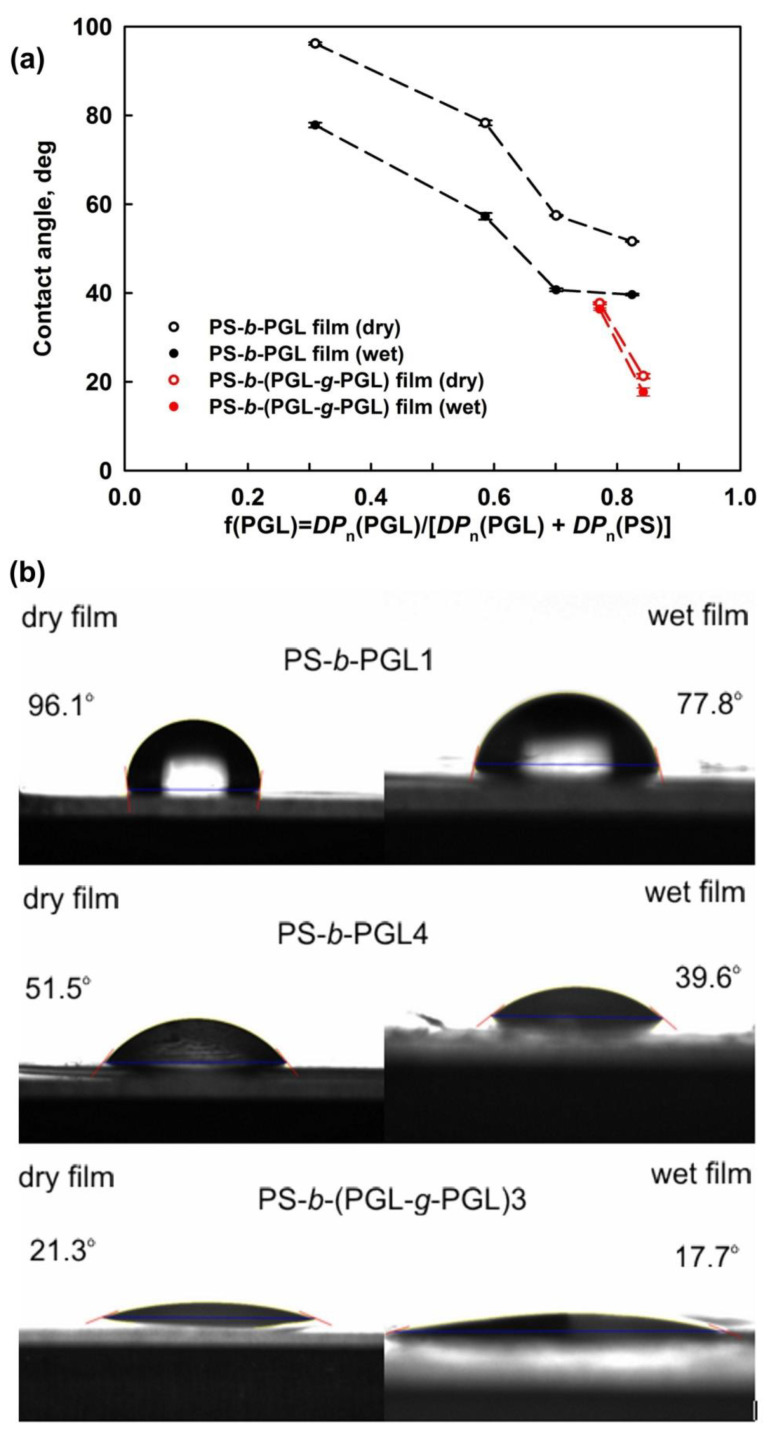
Static water contact angles measurements: (**a**) dependence of contact angles of water drops deposited on PS-*b*-PGL and PS-*b*-(PGL-*g*-PGL) copolymer films versus molar fraction of polyglycidol in copolymers chains. Error bars denote standard deviation; (**b**) water drops images on films from linear PS-*b*-PGL with the lowest (f(PGL) = 0.309) and the highest PGL fraction (f(PGL) = 0.824) and Coil-Brush PS-*b*-(PGL-*g*-PGL)3 copolymer with f(PGL) = 0.854.

Prior to static water contact angle measurements, the films were conditioned at room temperature in an environmental chamber. The incubation was carried out in air—at dry conditions (for 24 h) and in the atmosphere saturated with water vapor (for 48 h). Contact angle measurements were carried out immediately after taking out every film sample from the chamber. Dependences of wetting angle on molar fraction of PGL in copolymers and representative images of water drops deposited on films are shown in Figure 3a,b, correspondingly.

For linear amphiphilic copolymer “dry” films, the water contact angles decreased from ca. 96° for molar PGL fraction equal 0.309 to ca. 51° for copolymers with f(PGL) equal 0.824. The average water contact angles determined on the same films but stored in air saturated with water vapor were lower by several degrees, e.g., by 19° for f(PGL) = 0.309 and 12° for f(PGL) = 0.824. A different observation was made in the case of the PS-*b*-(PGL-*g*-PGL) copolymer dry films, for which the contact angles were significantly lower than for the films composed of linear blocks with similar PGL fraction. For instance, for Coil-Brush copolymer dry film with f(PGL) = 0.728 and f(PGL) = 0.854, the wetting angles were 37° and 21°, respectively. Furthermore, the conditioning of any Coil-Brush copolymer film in humidity chamber only slightly modified its wettability. In this case, water contact angles for the “wet” films were only 1–3 degrees lower than contact angles measured for the “dry” films. Presumably, the local packing of segments containing hydroxyl groups is higher in brush structures than in the linear ones; what makes water binding more effective due to hydroxyl groups compartmentalization and in result the removal of water is more difficult.

A similar tendency has been observed for hyperbranched polyglycidol grafted on silica surface [34].

In addition, the complete reproducibility of water contact angle values, determined for both linear and Coil-Brush films after each re-drying-re-wetting cycle, was noticed.

The water contact angle measurements revealed close relation between the fraction of hydrophilic PGL, the architecture of the PGL blocks in copolymers, the copolymer film appearance, and wettability.

One may also expect some qualitative relation between the wettability of copolymer films and copolymer micellization in aqueous media.

### 3.3. Determination of Copolymers Critical Micellization Concentration

CMC of copolymers was determined by spectrophotometry method and verified using dynamic light scattering method.

UV–vis measurements were conducted with 1,6-diphenylhexa-1,3,5-triene (DPH) as a probe. The hydrophobic-DPH is insoluble in water and gathers in micelles hydrophobic cores. Below CMC, in homogeneous water solutions of PS-*b*-(PGL-*g*-PGL), DPH is not soluble and such solutions do not absorb UV light. However, when concentration of PS-*b*-(PGL-*g*-PGL) is above CMC, the samples absorb UV light due to the accumulation of DPH in the hydrophobic core of micelles or aggregated fragments with increased hydrophobicity. The onset of aggregation was determined as the point of intersection of the tangents of the obtained curves and taken as the CMC (Figure S4). The results are given in Table 2.

As expected, PS-*b*-(PGL-*g*-PGL) Coil-Brush copolymers form aggregates above certain concentration. Studies show that the increase in the molar fraction of PGL resulted in the increase in CMC, indicating better solubility in water of copolymers with the higher PGL fraction.

In the next step, the behavior of PS-*b*-(PGL-*g*-PGL) copolymers in water was monitored by DLS. Measurements of solution/dispersion scattered light intensity were made for dialysates in water without UV probe. The obtained values were plotted as a function of copolymer concentration and, as in UV–vis, CMC was determined as the point of intersection of the tangents of the obtained curves (Figure S5). CMC values obtained by DLS method are in good agreement with those depicted on the basis of UV–vis.

**Figure 4:**
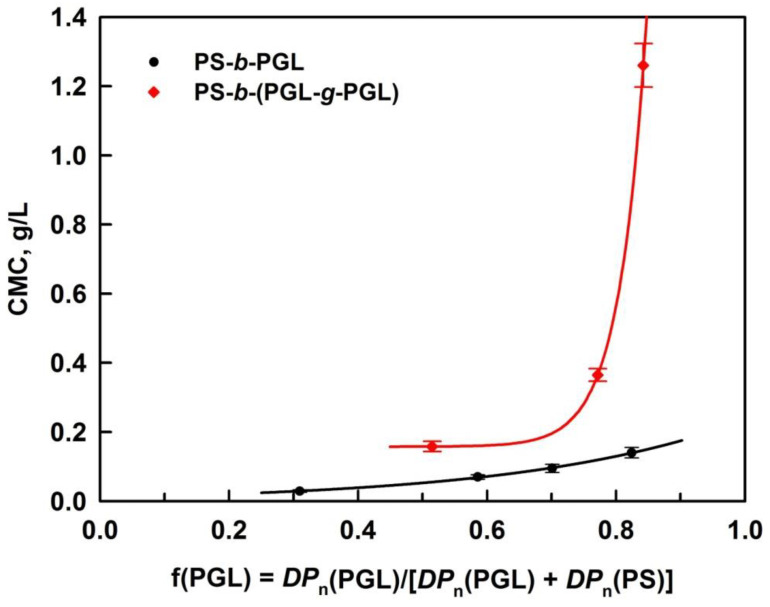
Dependence of CMC on the fraction of polyglycidol content (f(PGL)) in linear PS-*b*-PGL (black line) and Coil-Brush PS-*b*-(PGL-*g*-PGL) diblock copolymers (red line).

Studies show that regardless of the copolymer architecture, the increase in the molar fraction of PGL resulted in the increase in CMC, indicating better solubility of copolymers with the higher PGL fraction in water. It must be noted that CMC for PS-*b*-(PGL-*g*-PGL) was significantly higher than for PS-*b*-PGL and its rise with the increase in PGL fraction was much sharper, as shown in Figure 4.

### 3.4. Aggregation Process of PS-b-PGL and PS-b-(PGL-g-PGL) Copolymers

Dynamic light scattering studies were used also for determination of the size of particles formed in solution above CMC. Here, we compare aggregation of linear PS-*b*-PGL and Coil-Brush PS-*b*-(PGL-*g*-PGL) copolymers, size, and morphology of the formed self-assemblies. It was interesting to check whether there is any influence of the architecture of both “families” of PS/PGL block copolymers on the size and structure of the aggregates.

The average hydrodynamic diameters and diameter distributions determined for PS-*b*-PGL and PS-*b*-(PGL-*g*-PGL) copolymers by DLS method are given in Table 3. The hydrodynamic diameter distributions graphs for PS-*b*-(PGL-*g*-PGL) copolymers are shown in Figure S6.

Collected data indicate that self-assembly of the investigated Coil-Brush copolymers at concentrations above CMC yields particles with bimodal size distribution and with dispersity factor far higher than the typical one for monomodal polymeric micelles (<0.05). Analogous results were found for linear copolymers in previous study.

For particles prepared from PS-*b*-(PGL-*g*-PGL), the average diameters were in the range 8.5–14.5 nm, which corresponds to diameters of micelles. However, the intensity distributions are bimodal. The small population size is envisaged and accompanied by the large mode. The intensity average diameters were much larger, in a broad range from 103 to 172 nm, depending on the Coil-Brush copolymer composition. Their dispersity was also very large, however smaller than for linear copolymers. It is reasonable to assume that, at concentrations slightly above CMC, the dialysis of copolymers generates micelles, some of which aggregate. Presumably, the bigger clusters are formed due to weak stabilization of the PS(core)-PGL(shell) micelles in water. A similar observation was made in the case of particles prepared from the PS-*b*-PGL copolymers [32].

Results show that linear-brush copolymers formed smaller particles in solution and are less prone to formation of micellar clusters than their linear counterparts.

As mentioned, earlier studies of self-assembly of block copolymers, which differ with respect to macromolecular architecture but have the same composition, are seldom. The self-assembly of Coil-Brush polystyrene-*b*-poly[(4-vinylbenzyl chloride)-*co*-(styrene-*g*-poly(acrylic acid)] diblock copolymers with *M*_n_ = 115000 in dioxane-water (50/50 vol/vol) produced particles with bimodal size distribution, namely *D*_h_ = 84 nm and *D*_h_ = 830 nm. This size distribution suggests that the mixture of polymeric micelles and polymeric micelle aggregates were obtained [26].

**Figure 5:**
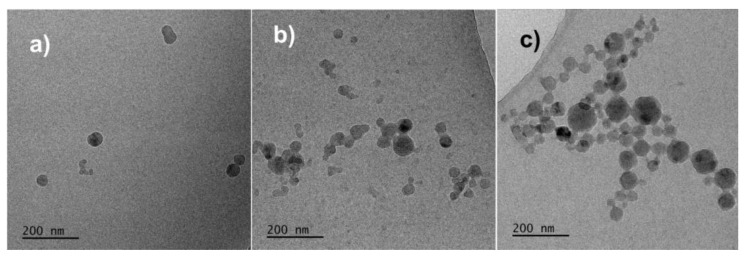
Representative cryo-TEM images of particles obtained by dialysis from: (**a**) PS-*b*-(PGL-*g*-PGL)1; (**b**) PS-*b*-(PGL-*g*-PGL)2; (**c**) PS-*b*-(PGL-*g*-PGL)3 block copolymers.

Results obtained in DLS studies were confirmed by cryo-TEM imaging (Figure 5). The images obtained from cryo-TEM revealed details of particles morphology. The size of particles obtained by dialysis of a set of PS-*b*-(PGL-*g*-PGL) copolymers was in the range 15–250 nm. There were visible individual spherical particles with the size in the range 10–50 nm and particles assemblies arranged into strings or irregular aggregates with the size up to 250 nm. TEM images for block PS-*b*-PGL copolymers presented in our previous paper [32] did not evidence of large amount of aggregates. The size of particle formed from these diblock copolymers was in the range of 24–55 nm.

## 4. Conclusions

An important result of the studies described in this paper is the positive verification of the hypothesis suggesting that PS-*b*-PGL copolymer with hydroxyls converted to potassium alkoxide groups can be used as macroinitiator enabling controlled anionic polymerization of 1-ethoxyethyl glycidyl ether (glycidol with blocked hydroxyls—GLB), which, after deblocking of hydroxyl groups, yield copolymers with polyglycidol grafts. The method was used for synthesis of PS-*b*-(PGL-*g*-PGL). However, it should also be suitable for the synthesis of polyglycidol containing copolymers with various types of structure. Thus, the developed method opens the way for the future studies of relations between copolymer architecture and its properties.

Studies of wettability of films prepared from the PS-*b*-PGL (hydrophobic coil–*b*–hydrophilic coil) and PS-*b*-(PGL-*g*-PGL) (hydrophobic coil–*b*-hydrophilic brush) copolymers the latter are much more hydrophilic (for the same molar fraction of polyglycidol). Moreover, the exposure of PS-*b*-PGL to water vapors increases film hydrophilicity. This effect was reversible, and films became less hydrophilic upon drying. The films prepared from PS-*b*-(PGL-*g*-PGL) were not sensitive to moisture and their drying at room temperature or exposure to water vapors did not much change their hydrophilicity. Apparently, the polyglycidol segments with brush architecture contain water molecules, which cannot be easily removed and maintain copolymer hydrophilicity. Thus, the hypothesis that the architecture of hydrophilic component of PS/PGL copolymers also has a strong influence on the self-assembly of copolymer macromolecules has been positively verified. The PS-*b*-(PGL-*g*-PGL) copolymers self-assemble much less efficiently than the PS-*b*-PGL copolymers with similar molar fraction of polyglycidol. Apparently, the brush type of polyglycidol part better stabilizes copolymer macromolecules in solution.

## Figures and Tables

**Table 1:** Characteristics of PS-OH oligomers, set of linear PS-*b*-PGL and PS-*b*-(PGL-*g*-PGL) copolymers.

Sample	*DP*_n_ of PS/PGL/gPGL Expected from Amount of Comonomer	*DP*_n_ of PS/PGL/gPGL Blocks in the Copolymer ^(c)^	f(PGL) ^(g)^	*M*_n_ (GPC) ^(d)^ g/mol	*M*_w_/*M*_n_	dn/dc, mL/g (Measured)	*DP*_n_ of PGL Side Block (GPC ^(f)^)
PS-OH1 ^(a)^	29/0	29/0	-	2800	1.013	0.1620 ^(e)^	-
PS-OH2 ^(a)^	29/0	29/0	-	2540	1.19	0.1620 ^(e)^	-
PS-*b*-PGL1 ^(b)^	29/12.7	29/13	0.309	3900	1.010	0.1340	-
PS-*b*-PGL2 ^(b)^	29/41.5	29/41	0.586	6100	1.030	0.1020	-
PS-*b*-PGL3 ^(b)^	29/68	29/68	0.701	8100	1.040	0.0870	-
PS-*b*-PGL4 ^(b)^	29/136	29/136	0.824	11,700	1.070	0.0720	-
PS-*b*-(PGL-*g*-PGL)1	29/12.7/2	29/13/2.2	0.515	5800	1.036	0.1156	1.78 (23.4 ^(h)^)
PS-*b*-(PGL-*g*-PGL)2	29/12.7/7	29/13/6.5	0.782	12,100	1.056	0.0939	7.57 (98.4 ^(h)^)
PS-*b*-(PGL-*g*-PGL)3	29/12.7/11	29/13/10.9	0.854	16,300	1.081	0.0880	11.40 (148.2 ^(h)^)

^(a)^ PS-OH1 and PS-OH2 denote hydroxyl terminated polystyrene blocks used for the synthesis of sets of linear PS-*b*-PGL (data from Ref. [32]) and PS-*b*-(PGL-*g*-PGL) (data in this work, see also Supplementary Materials) copolymers, respectively; ^(b)^ Data for linear PS-*b*-PGL copolymers taken from Ref. [32]; ^(c)^ *DP*_n_ calculated from ^1^H NMR spectra; ^(d)^ *M*_n_ determined from GPC chromatogram of copolymer (DMF solution and mobile phase); ^(e)^ dn/dc of polystyrene in DMF from literature data; ^(f)^ *DP*_n_ of side blocks in Coil-Brush copolymer determined by subtraction of molecular mass of the PS-*b*-PGL core from molecular mass of PS-*b*-(PGL-*g*-PGL) and dividing the result by molar mass of the glycidol unit in PGL (74.08 g) and the average number of –OH groups in PS-*b*-PGL1 (n = 14, taking into account that the PGL end-group contains two OH groups); ^(g)^ f(PGL) denotes molar fraction of polyglycidol units in copolymer, denoted as the ratio of the average number of polyglycidol units and the sum of the average number of polyglycidol and polystyrene units in PS-*b*-(PGL-*g*-PGL); ^(h)^ Total number of glycidol units in copolymer PGL block in the copolymer.

**Table 2:** CMC values of the set of PS-*b*-PGL diblock copolymers and PS-*b*-(PGL-*g*-PGL) Coil-Brush copolymers with various fractions of PGL units. Data for diblock copolymers taken from Ref. [32].

Copolymer Sample	f(PGL)	CMC, g/L (UV)	CMC, g/L (DLS)
PS-*b*-PGL1	0.309	-	0.029
PS-*b*-PGL2	0.586	-	0.070
PS-*b*-PGL3	0.701	-	0.096
PS-*b*-PGL4	0.824	-	0.140
PS-*b*-(PGL-*g*-PGL)1	0.515	0.141	0.158
PS-*b*-(PGL-*g*-PGL)2	0.782	0.541	0.365
PS-*b*-(PGL-*g*-PGL)3	0.854	1.23	1.26

**Table 3:** Hydrodynamic diameters of particles formed by self-assembly of PS-*b*-PGL (data taken from Ref. [32]) and PS-*b*-(PGL-*g*-PGL) copolymers determined by DLS. *D*_h,n_ and *D*_h,si_ denote number and scattering intensity average diameters and scattering intensity average dispersity, respectively.

Copolymer Sample	Copolymer Conc., g/L	*D*_h,n_, nm	*D*_h,si_, nm	Dispersity _SM_
PS-*b*-PGL1	0.12	27.3	27.3/127	0.38
PS-*b*-PGL2	0.19	49.4	49.4/165	0.41
PS-*b*-PGL3	0.24	50.7	50.7/187	0.55
PS-*b*-PGL4	0.26	102	102/239	0.70
PS-*b*-(PGL-*g*-PGL)1	0.38 0.75	14.5 8.5	14.5/123 8.5/172	0.265 0.224
PS-*b*-(PGL-*g*-PGL)2	0.36 0.60	8.5 14.5	8.5/154 14.5/136	0.204 0.264
PS-*b*-(PGL-*g*-PGL)3	1.86	14.0	14.0/129	0.285

## Data Availability

The data presented in this study are available on request from the corresponding author.

## Supplementary Materials

The following supporting information can be downloaded at: https://www.mdpi.com/article/10.3390/polym14020253/s1: Figure S1. ^1^H NMR spectrum (in CDCl_3_) of PS-*b*-(PGL-*g*-PGLB)1 copolymer. Figure S2. FTIR IR spectra of a set of PS-*b*-(PGL-*g*-PGL) copolymers. Figure S3. GPC traces of PS-OH macroinitiator and linear PS-*b*-PGL diblock copolymer. Analysis performed in DMF, at 45 °C. Figure S4. Determination of CMC by spectrophotometric method for (a) PS-*b*-(PGL-*g*-PGL)1 (b) PS-*b*-(PGL-*g*-PGL)2 and (c) PS-*b*-(PGL-*g*-PGL)1. Figure S5. Determination of CMC by DLS method for (a) PS-*b*-(PGL-*g*-PGL)1 (b) PS-*b*-(PGL-*g*-PGL)2 and (c) PS-*b*-(PGL-*g*-PGL)3. Figure S6. The hydrodynamic diameter distributions of micelles and their aggregates for (a) PS-*b*-(PGL-*g*-PGL)1 with concentration 0.75 g/L, (b) PS-*b*-(PGL-*g*-PGL)1 with concentration 0.38 g/L, (c) PS-*b*-(PGL-*g*-PGL)2 with concentration 1.36 g/L, (d) PS-*b*-(PGL-*g*-PGL)2 with concentration 0.60 g/L, (e) PS-*b*-(PGL-*g*-PGL)3 with concentration 1.86 g/L, (f) PS-*b*-(PGL-*g*-PGL)3 with concentration 0.18 g/L. Figure S7. AFM Pictures used for determination of RMS parameters characterizing roughness of copolymer films. Values of RMS parameters are given on the pictures.
