# Supplementary material for: Synthesis, Hydrophilicity and Micellization of Coil-Brush Polystyrene-b-(polyglycidol-g-polyglycidol) Copolymer—Comparison with Linear Polystyrene-b-polyglycidol
Source: Polymers (Basel). 2022 Jan 8;14(2):253. doi: 10.3390/polym14020253 (PMC8778311; doi:10.3390/polym14020253)
Supplement: Supplementary file 1 [file polymers-14-00253-s001.zip › polymers-1497019-supplementary.pdf]

Supporting Materials

Synthesis, hydrophilicity and micellization of coil-brush polystyrene-*b*-(polyglycidol-*g*-polyglycidol) copolymer – comparison with linear polystyrene-*b*-polyglycidol

Mariusz Gadzinowski, Maciej Kasprów, Teresa Basinska, Stanislaw Slomkowski, Łukasz Otulakowski, Barbara Trzebicka , and Tomasz Makowski

SM1 Preparation of PS-*b*-PGL block copolymer.

Synthesis of PS-*b*-PGL consisted of a) synthesis of polystyrene terminated with hydroxyethyl group (PS-OH), b) anionic polymerization of glycidol with blocked hydroxyl group (GLB) initiated with PS-OK macroinitiator (produced in reaction of PS-OH with potassium mirror) and c) deprotection of GLB units in PS-*b*-PGLB copolymer.

(a) Synthesis of polystyrene terminated with hydroxyl group (PS-OH).

Polystyrene with targeted molar mass M_n = 3000 g/mol (degree of polymerization 29) was synthesized by anionic polymerization initiated with sec-butyllithium. Solution of 48.5 g (0.46 mol) of dry styrene in dry toluene was prepared in deaerated ampoule and subsequently, 11.5 mL of 1.4 mol/L initiator solution (0.016 mol) was added. The mixture was stirred at 0-5°C for 1h. The reaction vial was then opened in argon atmosphere and an excess of ethylene oxide was added (about 10 mL). After 15 min. an excess of formic acid in THF (15 mL 10%) was added to terminate active chains ends transforming them into hydroxyl groups. The resulting hydroxyl-terminated polystyrene (PS-OH) was precipitated into methanol and dried in vacuum.

Analysis:

^{1}H-NMR: M_n PS-OH=3090 g/mol total (29 styrene units + C_4H_9 +OH)

GPC RI: M_n PS-OH=2540, M_w/M_n=1.19

(b) Preparation of diblock polystyrene-*b*-poly(ethoxyethyl glycidyl ether) copolymer.

Linear polystyrene-*b*-(1-ethoxyethyl glycidyl ether) copolymer (PS-*b*-PGLB) was prepared by polymerization of GLB using PS-macroinitiator. The macroinitiator was prepared by dissolving PS-OH (19.9 g, 0.00644 mol) in 125 mL of dry THF and reacting it for 24 h with freshly prepared potassium mirror. The resulting solution was poured into next vial and 12.91 g (0.0884 mol) of GLB was added. Polymerization was carried out at 45 °C for 4 days. Then, active centers were terminated by adding excess of formic acid solution. The resulting

copolymer was precipitated into warm water. The crude viscous product was dried in vacuum, dissolved in 1,4-dioxane and precipitated into methanol.

Theoretical values: $DP_{n,PGLB}$=13.7, $M_{n,PS\text{-}PGLB}$=5090 g/mol

Analysis:

^{1}H-NMR: $M_{n,PS\text{-}PGLB}$=5000, $DP_{n,PS}$ = 29, $DP_{n,GLB}$ = 13

GPC RI: $M_{nPS\text{-}PGLB}$=5120, M_w/M_n=1.13

(c) Deprotection of GLB units in PS-*b*-PGLB linear copolymer.

Deprotection of the 1-ethoxyethyl glycidyl ether units in copolymer was performed in the following way: 32.0 g of PS-*b*-PGLB was dissolved in 5 mL of 1,4-dioxane and 10 mL of methanol was added dropwise until the mixture become slightly opaque. Then 11 mg of $AlCl_3 \cdot 6H_2O$ was added and the mixture was stirred at 40°C for 3h. The copolymer was dialyzed against 10^{-2} mol/l HCl and subsequently against water using SERVA SpectraPor dialyzing tube with MWCO (molar mass cut-off) 1000 g/mol. The final product was lyophilized. Obtained sample was denoted PS-*b*-PGL1.

SM2 Characterization of intermediates of synthesized diblock copolymers.

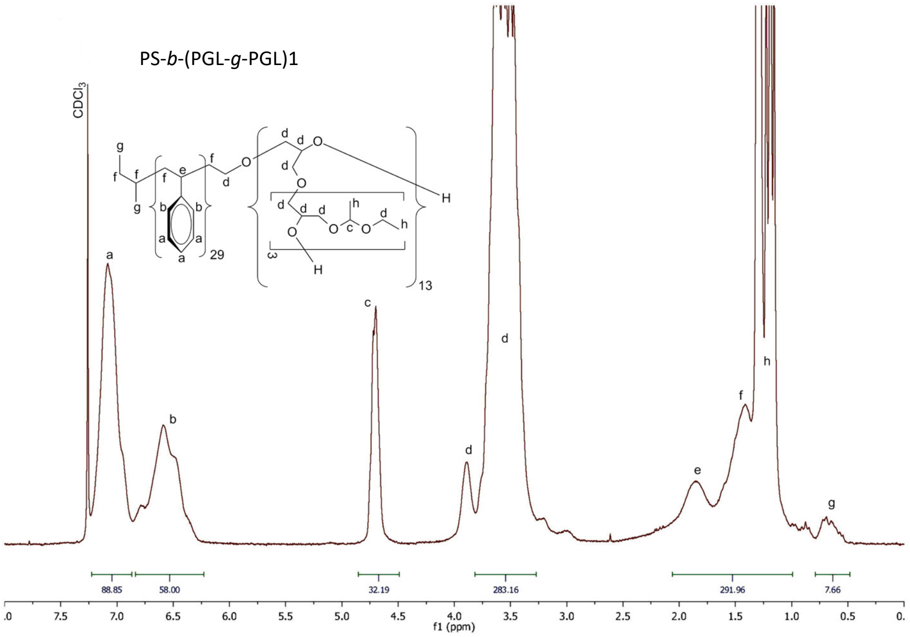

Figure S1. ^{1}H NMR spectrum (in $CDCl_3$) of PS-*b*-(PGL-*g*-PGLB)1 copolymer.

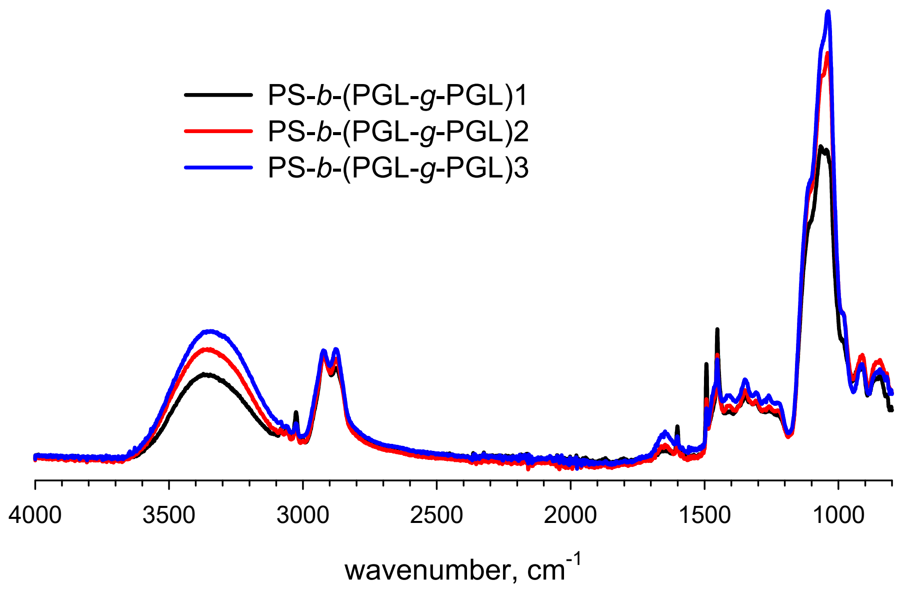

Figure S2. FTIR IR spectra of a set of PS-*b*-(PGL-*g*-PGL) copolymers.

3600-3050 cm^{-1} O-H stretching intermolecular bonded,

3000-2750 cm^{-1} aliphatic C-H stretching,

1650 cm^{-1} aromatic C-H bending,

1500-1200 cm^{-1} $-CH_2-$ bending and O-H bending,

1130-1000 cm^{-1} C-O stretching primary alcohol.

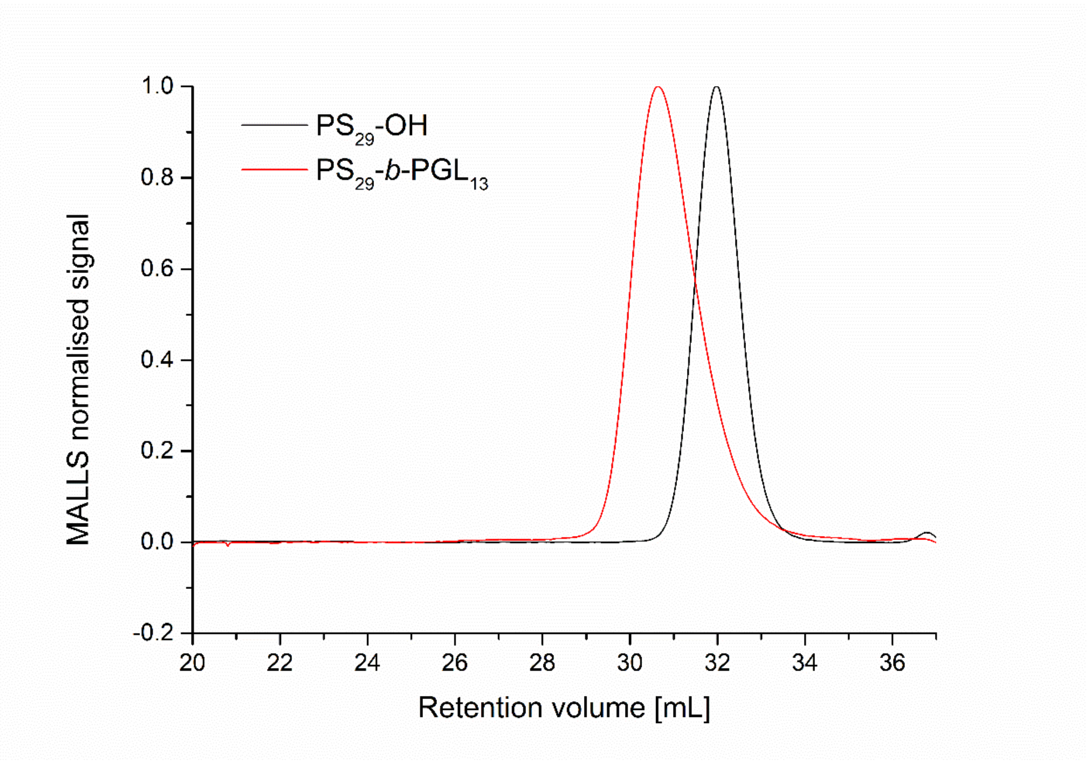

Figure S3. GPC traces of PS-OH macroinitiator and linear PS-*b*-PGL diblock copolymer. Analysis performed in DMF, at 45 °C.

SM3 Plots used for determination of CMC of PS-*b*-(PGL-*g*-PGL)1, PS-*b*-(PGL-*g*-PGL)2 and PS-*b*-(PGL-*g*-PGL)3 copolymers

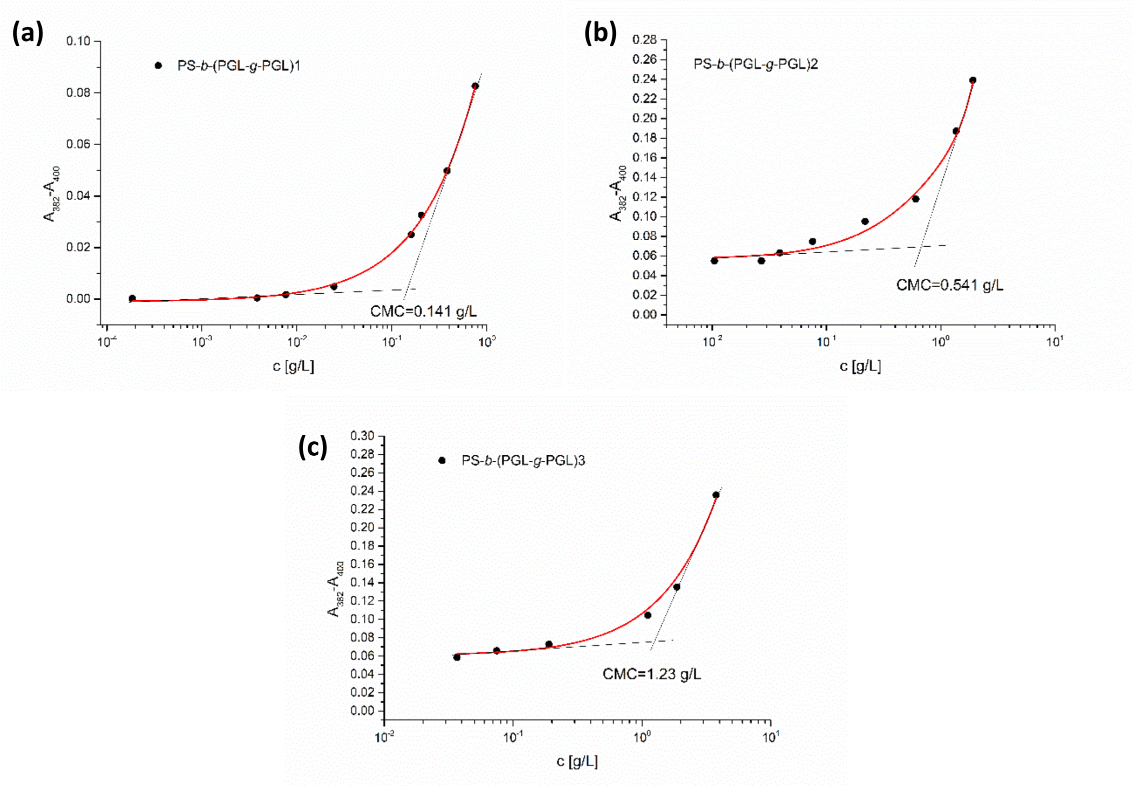

Figure S4. Determination of CMC by spectrophotometric method for a) PS-*b*-(PGL-*g*-PGL)1 b) PS-*b*-(PGL-*g*-PGL)2 and c) PS-*b*-(PGL-*g*-PGL)1.

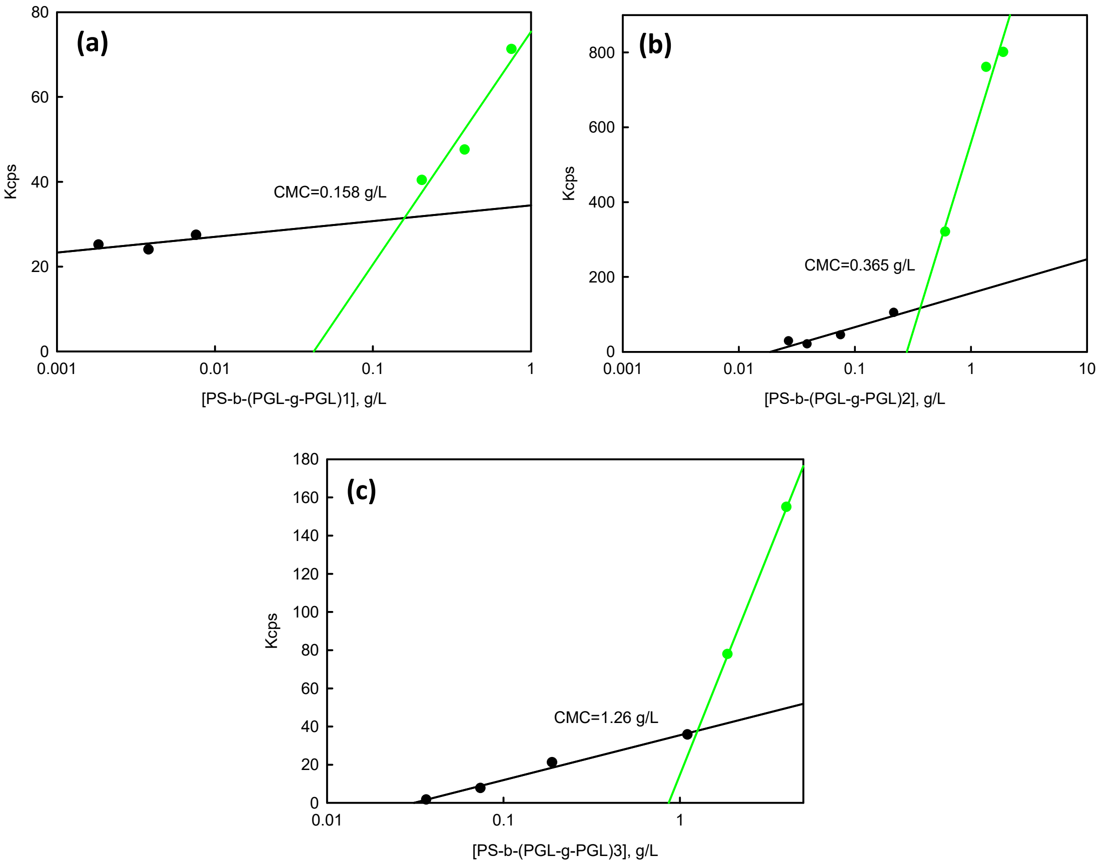

Figure S5. Determination of CMC by DLS method for a) PS-*b*-(PGL-*g*-PGL)1 b) PS-*b*-(PGL-*g*-PGL)2 and c) PS-*b*-(PGL-*g*-PGL)3.

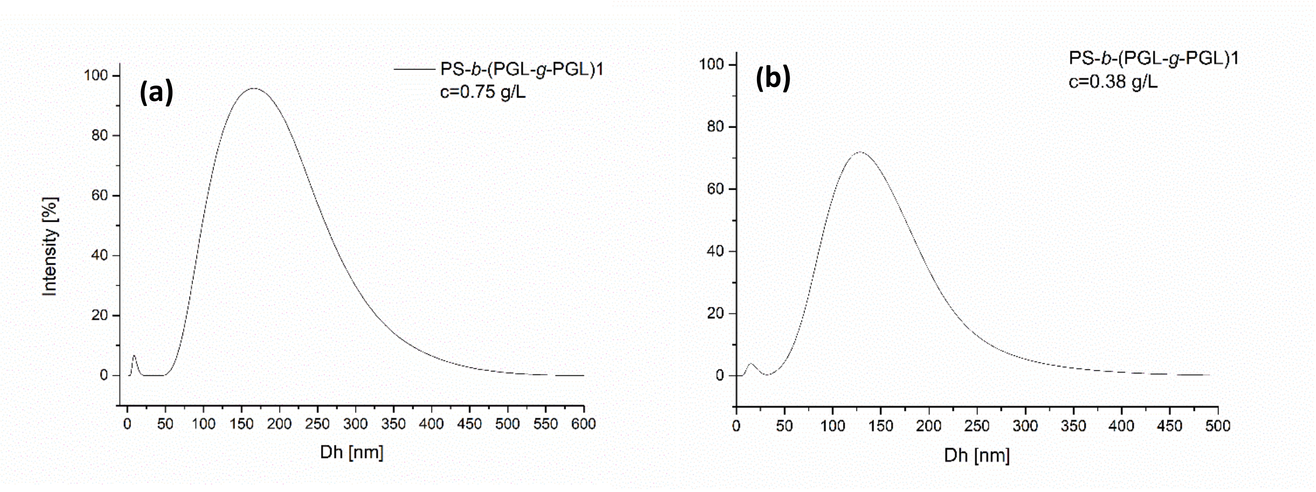

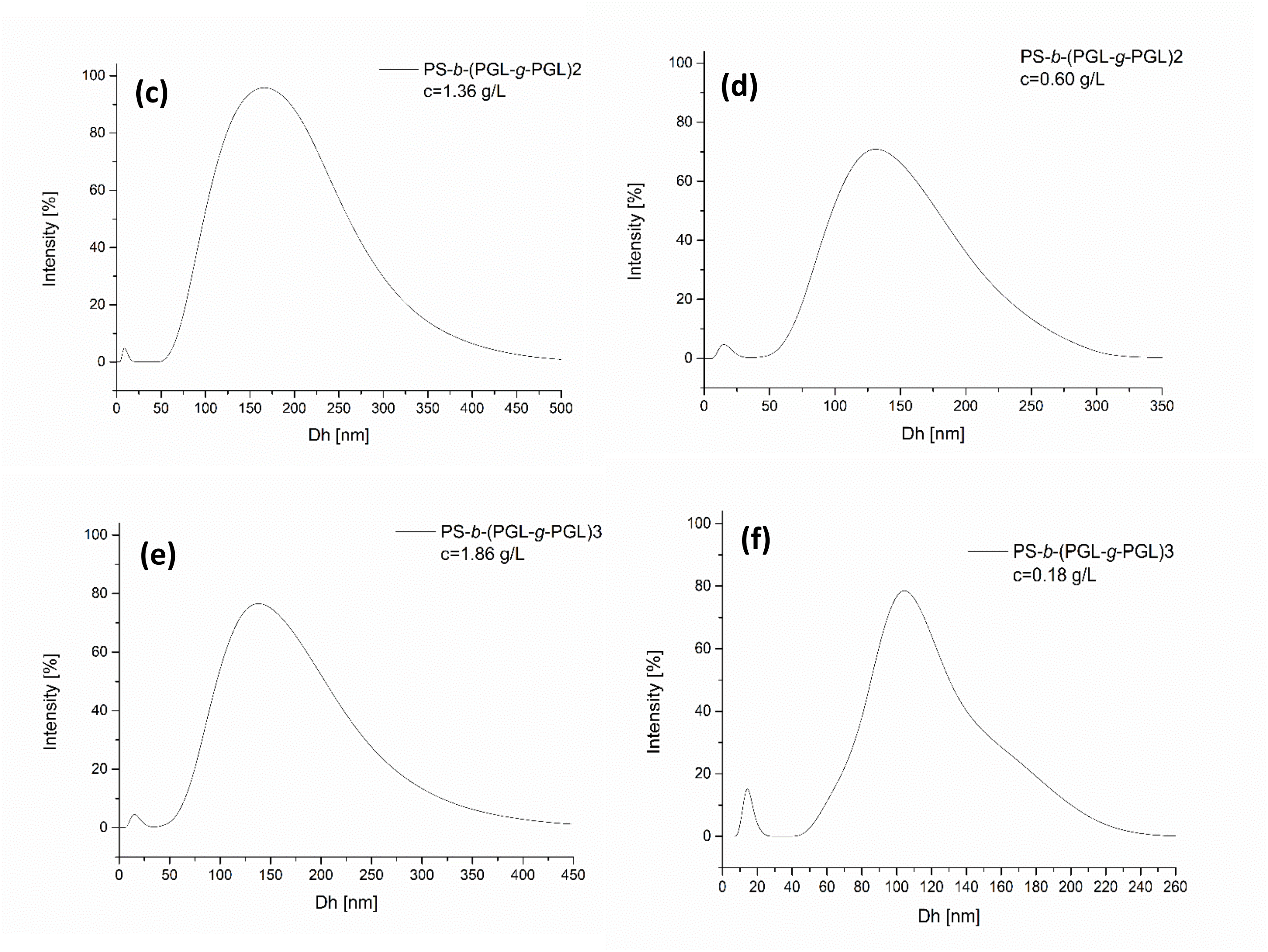

Figure S6. The hydrodynamic diameter distributions of micelles and their aggregates for (a) PS-*b*-(PGL-*g*-PGL)1 with concentration 0.75 g/L, (b) PS-*b*-(PGL-*g*-PGL)1 with concentration 0.38 g/L, (c) PS-*b*-(PGL-*g*-PGL)2 with concentration 1.36 g/L, (d) PS-*b*-(PGL-*g*-PGL)2 with concentration 0.60 g/L, (e) PS-*b*-(PGL-*g*-PGL)3 with concentration 1.86 g/L, (f) PS-*b*-(PGL-*g*-PGL)3 with concentration 0.18 g/L.

SM4 AFM Pictures used for determination of roughness of films prepared from PS-*b*-PGL2, PS-*b*-PGL4, PS-*b*-(PGL-*g*-PGL)1, PS-*b*-(PGL-*g*-PGL)3 copolymers

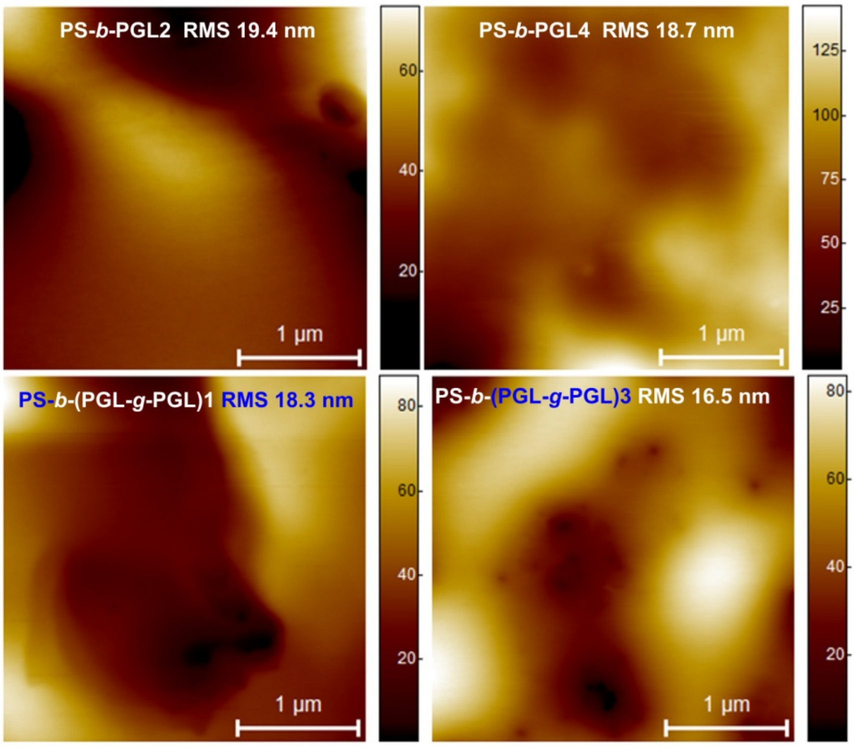

Figure S7 AFM Pictures used for determination of RMS parameters characterizing roughness of copolymer films. Values of RMS parameters are given on the pictures.
